# Design of health information management model for elderly care using an advanced higher-order hybrid clustering algorithm from the perspective of sports and medicine integration

**DOI:** 10.1371/journal.pone.0302741

**Published:** 2024-05-17

**Authors:** Ning Zhao, Wenkai Zhao, Xiaoliang Tang, Chuanming Jiao, Zhong Zhang

**Affiliations:** 1 Physical Education Department, Qiqihar Medical University, Qiqihar, Heilongjiang, China; 2 The Third Affiliated Hospital of Qiqihar Medical University, Qiqihar, Heilongjiang, China; Cyprus International University Faculty of Engineering: Uluslararasi Kibris Universitesi Muhendislik Fakultesi, TURKEY

## Abstract

In the context of integrating sports and medicine domains, the urgent resolution of elderly health supervision requires effective data clustering algorithms. This paper introduces a novel higher-order hybrid clustering algorithm that combines density values and the particle swarm optimization (PSO) algorithm. Initially, the traditional PSO algorithm is enhanced by integrating the Global Evolution Dynamic Model (GEDM) into the Distribution Estimation Algorithm (EDA), constructing a weighted covariance matrix-based GEDM. This adapted PSO algorithm dynamically selects between the Global Evolution Dynamic Model and the standard PSO algorithm to update population information, significantly enhancing convergence speed while mitigating the risk of local optima entrapment. Subsequently, the higher-order hybrid clustering algorithm is formulated based on the density value and the refined PSO algorithm. The PSO clustering algorithm is adopted in the initial clustering phase, culminating in class clusters after a finite number of iterations. These clusters then undergo the application of the density peak search algorithm to identify candidate centroids. The final centroids are determined through a fusion of the initial class clusters and the identified candidate centroids. Results showcase remarkable improvements: achieving 99.13%, 82.22%, and 99.22% for F-measure, recall, and precision on dataset S1, and 75.22%, 64.0%, and 64.4% on dataset CMC. Notably, the proposed algorithm yields a 75.22%, 64.4%, and 64.6% rate on dataset S, significantly surpassing the comparative schemes’ performance. Moreover, employing the text vector representation of the LDA topic vector model underscores the efficacy of the higher-order hybrid clustering algorithm in efficiently clustering text information. This innovative approach facilitates swift and accurate clustering of elderly health data from the perspective of sports and medicine integration. It enables the identification of patterns and regularities within the data, facilitating the formulation of personalized health management strategies and addressing latent health concerns among the elderly population.

## Introduction

In the contemporary landscape, health emerges as a pivotal facet in the quest for an improved quality of life. Nevertheless, the escalating prominence of health issues stemming from inadequate physical activity poses a substantial menace to well-being. Of particular concern is the surge in chronic ailments among the elderly, burdening healthcare services and precipitating a public health crisis. To combat these challenges, the government has initiated a strategy that amalgamates sports and medicine—melding medical expertise with sports science—to employ exercise and fitness as preventative and therapeutic measures against diseases.

The overarching objective of this integration is to optimize both physical and mental well-being by leveraging exercise, sports, and fitness to enhance health and stave off diseases. This approach enables the real-time assessment of health metrics during physical activity, facilitating the formulation of tailored exercise regimens for the elderly. These programs aim to bolster physical health, mitigate disease risks, and elevate their overall quality of life. As data volumes burgeon, manual management proves increasingly inefficient. Artificial intelligence (AI) methodologies offer a solution by enabling efficient and precise analysis, processing, and prognostication of data. Leveraging advanced sensor, computer, and internet technologies holds the promise of elevating the intelligence quotient of community medical and health services. The focal point of ongoing research revolves around utilizing body-health integration data to cater to the specific medical and health needs of the elderly.

Recent advancements in scholarly research have revealed breakthroughs in clustering algorithms [1], particularly in the domain of unsupervised learning. These algorithms adeptly handle diverse data types—continuous, discrete, ordered, or nominal—alongside large datasets. Automating the determination of optimal cluster numbers and outcomes eliminates the need for human intervention. This ensures that similar entities or data points coalesce, promoting intra-group homogeneity and inter-group heterogeneity, thereby enhancing the efficient management of elderly health data. Concurrently, researchers have engaged in sensor-based somatosensory data collection, expanding the technological toolkit for health data management within the body-medicine integration paradigm. However, challenges persist in elderly health data management using clustering algorithms within the body-medicine nexus. Notably, the sensitivity of clustering algorithms to initial centroid selection poses a challenge, potentially leading to suboptimal clustering outcomes. Additionally, as unsupervised learning methods, clustering algorithms often involve non-convex functions with multiple local extrema in their objective functions, increasing susceptibility to converging on local optima [2].

To address these concerns, the Particle Swarm Optimization (PSO) algorithm [3] emerges as a powerful global optimization technique, mimicking natural bird foraging to navigate complex data spaces. Recent applications underscore its effectiveness in resolving clustering predicaments. Nevertheless, inherent in existing PSO algorithms is the risk of converging toward local optima, necessitating further exploration and refinement.

This paper tackles the aforementioned challenges by optimizing the PSO algorithm, treating clustering as an optimization problem. Utilizing an intelligent optimization algorithm enhances the stability of the clustering process. To this end, a high-order hybrid clustering algorithm is proposed to design a comprehensive health information management model for elderly individuals within the realm of physical medicine integration. The primary contributions of this study are outlined below:

1. Enhancement of the PSO algorithm: Introducing a Weighted Covariance Matrix-based Global Evolution Dynamic Model (GEDM) refines the depiction of population distribution by employing weighted covariance calculations. Strategic utilization of GEDM or the conventional PSO algorithm at distinct stages strengthens algorithmic adaptability and resilience, augmenting convergence speed and preventing entrapment in local optima.
2. Development of a higher-order hybrid clustering algorithm: This approach integrates the peaking algorithm with the PSO clustering algorithm. Initially, the PSO clustering algorithm iteratively forms class clusters, followed by applying the density peaking search algorithm to ascertain candidate centroids based on data attributes. The final centroids are derived by amalgamating the initial class clusters with the identified candidate centroids.
3. Efficient clustering of elderly health text information using the higher-order hybrid clustering algorithm with the LDA topic vector model: Employing this algorithm for text clustering on a synthetic dataset, utilizing three distinct text vector formation methods—Vector Space Model (VSM), Doc2vec, and LDA Topic Model—underscores the efficacy of the LDA Topic Model-based approach for optimal text clustering within elderly health data analysis.

In this paper, the current state of the art of clustering algorithms and the current state of application of PSO algorithms nowadays will be presented in Section 2. The improved PSO algorithm constructed in this paper and the higher order hybrid clustering algorithm based on density values and the improved PSO algorithm are presented in Section 3. Section 4 focuses on describing the experimental results and discussing the performance of the scheme, comparing and analysing it with the classical scheme, and conducting ablation experiments to explore the role of each module of the model. Finally, it also explores the clustering of text data by this paper’s higher-order hybrid clustering algorithm under different text vector formation methods to improve the management of health data of the elderly in the context of physical-medical integration. Section 5 concludes with a summary discussing the performance of the higher-order hybrid clustering algorithm constructed in this paper and its application to elderly health data management.

## Related works

### Hybrid clustering algorithm

The K-Means algorithm, well-known for its simplicity, has limitations that impede its ability to produce flawless clustering results [4]. In response, literature [5] introduced the Fuzzy C-Means (FCM) algorithm, allowing points to belong to multiple clusters, a departure from K-Means’ strict partitioning. However, sensitivity to initial values persisted, leading to subsequent enhancements. The peak method proposed in literature [6] was employed to estimate cluster centers for initial partitioning, while literature [7] advocated adjusting proximity distances to fortify FCM against outliers. Further advancements aimed to enhance clustering algorithms’ efficacy. Literature [8] presented a probabilistic perspective on clustering, extracting data objects from specific probability distributions and assuming the overall data distribution as a blend of several distributions. Similarly, literature [9] showcased Density-Based Spatial Clustering of Applications with Noise (DBSCAN), a density-based algorithm capable of identifying arbitrarily shaped clusters without constraints, demonstrating robust performance on sizable datasets. Building upon DBSCAN, literature [10] introduced density peak clustering, derived from the DBSCAN algorithm, excelling in discerning non-spherical clusters and accurately delineating intricate data distributions.

These algorithms face challenges related to initial clustering center selection and the tendency to converge toward local optima, potentially hindering the attainment of globally optimal clustering outcomes. Recent research has focused on leveraging PSO-based data clustering to devise hybrid clustering algorithms, combining PSO with conventional methods to elevate clustering efficacy [11]. These PSO-based algorithms dynamically fine-tune parameters like inertia weights, using population fitness variance to ascertain the convergence timing of PSO and K-Means algorithms [12]. Additionally, they incorporate real-time monitoring of optimal values within individual particles and particle clusters, promptly executing mutation operations on prematurely converging particles to explore globally optimal initial clustering centers for K-Means algorithms.

Hybrid clustering algorithms provide a more comprehensive understanding of data, combining the global search and optimization capabilities of PSO with the expertise of traditional clustering algorithms tailored to specific data structures and features. Literature [13] introduces the PSO-K-means algorithm, which initializes a particle in the particle swarm using clustering results derived from K-Means. Alternatively, literature [14] presents a hybrid algorithm that combines PSO and K-Means, utilizing PSO for global search at the optimization’s outset and leveraging K-Means for accelerated convergence near the optimal solution. In addressing nonlinear division clustering challenges, literature [15] pioneers a hybrid clustering algorithm that integrates fuzzy adaptive PSO and K-Means methodologies.

### PSO optimization model

To enhance the effectiveness of hybrid clustering algorithms, researchers have worked to refine both clustering and PSO methodologies. Literature [16] introduces the Evolutionary Particle Swarm Optimization (EPSO) algorithm, rooted in particle swarm evolution. This approach initiates particles uniformly across the input data space, with subsequent generations dynamically adjusting to pursue optimal positions. However, relying on inter-particle information exchange limits its ability to break free from local optima, curtailing performance on intricate problems and constraining global search capability.

In pursuit of reduced computational complexity, literature [17] proposes an enhanced mPSC (Particle Swarm Clustering) algorithm, aiming to streamline PSC for improved efficiency with large-scale datasets. Despite simplifying computational processes and reducing manual input parameters, mPSC faces challenges related to dataset characteristics and initial parameter choices. These challenges result in susceptibility to local optima, diminished convergence efficiency, and compromised clustering accuracy, highlighting the need for more comprehensive optimization. Literature [18] introduces a PSC-RCE algorithm, incorporating Rapid Centroid Estimation (RCE) [19] to simplify PSC’s update rules. This approach, termed PSC-RCE, aims to streamline the clustering process. While PSO-based clustering mitigates the risk of local optima, challenges persist. The update mechanism in PSO algorithms, reliant on historical optimal solutions, leads to diminished convergence efficiency during initial search stages and a subsequent decline in population diversity. This inefficiency poses a substantial challenge, particularly in scenarios involving large-scale datasets and stringent real-time requirements, due to prolonged convergence times and heightened computational resource consumption.

The PSO algorithm encounters limitations when handling high-dimensional, intricate shapes, and noisy data, often yielding clustering outcomes lacking accuracy and stability. These limitations directly impact the algorithm’s reliability and practical utility, constraining its applicability in real-world scenarios. Consequently, this paper aims to enhance the PSO methodology by integrating it with density algorithms, forging a higher-order hybrid clustering algorithm. The objective is to craft a health data management model tailored for the elderly within the framework of physical-medical integration.

### Model design

**Fig 1:**
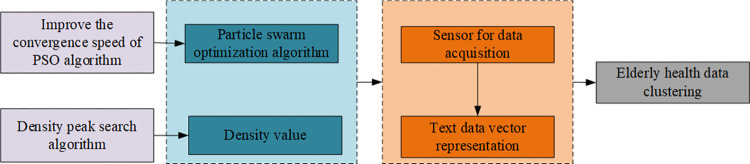
Model frame drawing.

The model diagram in Fig 1 illustrates the framework for elderly health information management based on the higher-order hybrid clustering algorithm developed in this paper. Our approach involves refining the PSO algorithm and integrating density values to construct an efficient higher-order hybrid clustering algorithm. This algorithm processes sensor-collected data, representing it in vector form, and concludes with the effective clustering management of elderly health data.

### Optimization of PSO

Within the n-dimensional search space of the PSO algorithm, each particle is characterized by two vectors: the position vector (*P*) and the velocity vector (*V*). Initially, every particle possesses randomized initial velocity and position values. The particle’s position denotes a potential solution within the problem space. Throughout the search phase, the PSO algorithm iteratively updates particle information. Let the individual history optimal solution *pbest*_*i*_ for particle i and the global history optimal solution *gbest*_*i*_ for the whole population be represented as follows:

$$pbest_{i}=[p_{i,1},p_{i,2},\ldots ,p_{i,n}]$$ (1)

$$gbest_{i}=[g_{i,1},g_{i,2},\ldots ,g_{i,n}]$$ (2)

When the particle moves in the search space, the velocity *v*_*id*_ and position *x*_*id*_ of each particle i are updated according to the Formulas (3) and (4).


$$v_{id}(t+1)=w*v_{id}(t)+c1*rand_{1}(pbest_{id}-x_{id}(t))+c2*rand_{2}*(gbest_{d}-x_{id}(t))$$ (3)

$$x_{id}(t+1)=x_{id}(t)+v_{id}(t+1)$$ (4)

Where *w* denotes the inertia weights, *c*1 and *c*2 denote the acceleration constants, *v*_*id*_(*t*) denotes the velocity of the ith particle in the dth dimension at the t-th iteration, and rand1 and rand2 are random numbers in the range [0,1]. It is established that the efficacy of PSO is heightened as the inertia weights progressively decrease in tandem with the number of iterations. The subsequent representation illustrates the linear descent of inertia weights:

$$w=w_{\max}-(w_{\max}-w_{\min})*\frac{t}{t_{\max}}$$ (5)

**Fig 2:**
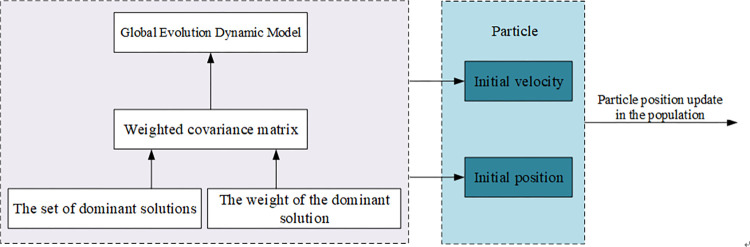
Weighted covariance matrix based GEDM model.

To further enhance the convergence efficacy of the PSO algorithm and mitigate local optima, our investigation reveals EDA [20] as a robust evolutionary algorithm. EDA estimates population evolution through probabilistic model sampling and learning, exhibiting exceptional performance in addressing intricate optimization problems. Within this framework, GEDM [21] assumes a pivotal role as a core constituent of EDA. Our decision was to integrate GEDM into the PSO algorithm, depicted in Fig 2. This integration involves constructing a GEDM model utilizing a weighted covariance matrix. This approach enables a more precise depiction of the population’s distribution through weighted covariance matrix calculations, thereby furnishing the algorithm with an enhanced estimation of the evolutionary trajectory. The computational methodology for the GEDM model based on the weighted covariance matrix is delineated below:

$$w_{i}=\frac{\ln(m+1)-\ln(i)}{\sum_{i=1}^{m}\left(\ln(m+1)-\ln(i)\right)}$$ (6)

$$X{\left(t\right)}_{mean}=\sum_{i=1}^{m}w_{i}*X_{i}(t)$$ (7)

$$Cov(t)=\frac{1}{m-1}*\sum_{i=1}^{m}(X_{i}(t)-X{\left(t\right)}_{mean})*{\left(X_{i}(t)-X{\left(t\right)}_{mean}\right)}^{T}$$ (8)

We set m to denote the number of solutions selected as dominant, and {*X*_1_,*X*_2_,*X*_3_,⋯,*X*_*m*_} denotes the set of dominant solutions, where *X*_1_ denotes the optimal solution. *w*_*i*_ denotes the weight of the ith dominant solution, the higher the ranking of the solution the greater the corresponding weight.

*Cov*(*t*) represents the weighted covariance matrix of the dominant solutions in the proposed model. When using GEDM to update the population information, we use the Formula (9) to update the position of particles in the population.


$$X_{i}(t+1)=Gaussian(X{\left(t\right)}_{mean},Cov(t))+rand*(X{\left(t\right)}_{mean}-X_{i}(t))$$ (9)

To effectively merge GEDM and PSO, a selection process determines whether GEDM or PSO updates the population information at various stages of the optimization process, guided by distinct probabilities. During the initial phase, GEDM receives a higher probability for updating population information. This prioritization leverages the strengths of advantageous particles to ascertain a superior evolutionary trajectory, thereby accelerating the convergence rate. Conversely, in the latter stages of optimization, PSO garners a higher probability for updating population information. This strategic shift capitalizes on PSO’s robust local search capabilities to enhance convergence accuracy. The probability model governing the selection between GEDM and PSO to update population information is depicted below:

$$y=y_{\max}-(y_{\max}-y_{\min})*\frac{t}{t_{\max}}$$ (10)

Where *y*_max_ and *y*_min_ denote the maximum and minimum probability of selecting GEDM to update population information, respectively.

### Higher order hybrid clustering algorithm

Drawing from the density peak clustering method established in literature [9, 10], this approach adeptly manages clusters of varying shapes, identifies noise points within the data for exclusion from clustering, and offers an accurate depiction of data distribution. To enhance clustering precision, we introduce the density peak algorithm into the hybrid clustering framework based on the PSO algorithm in this section, thereby constructing a higher order hybrid clustering algorithm for performance enhancement. The PSO algorithm utilized within this higher-order framework is the refined PSO algorithm outlined in Section 3.1.

**Fig 3:**
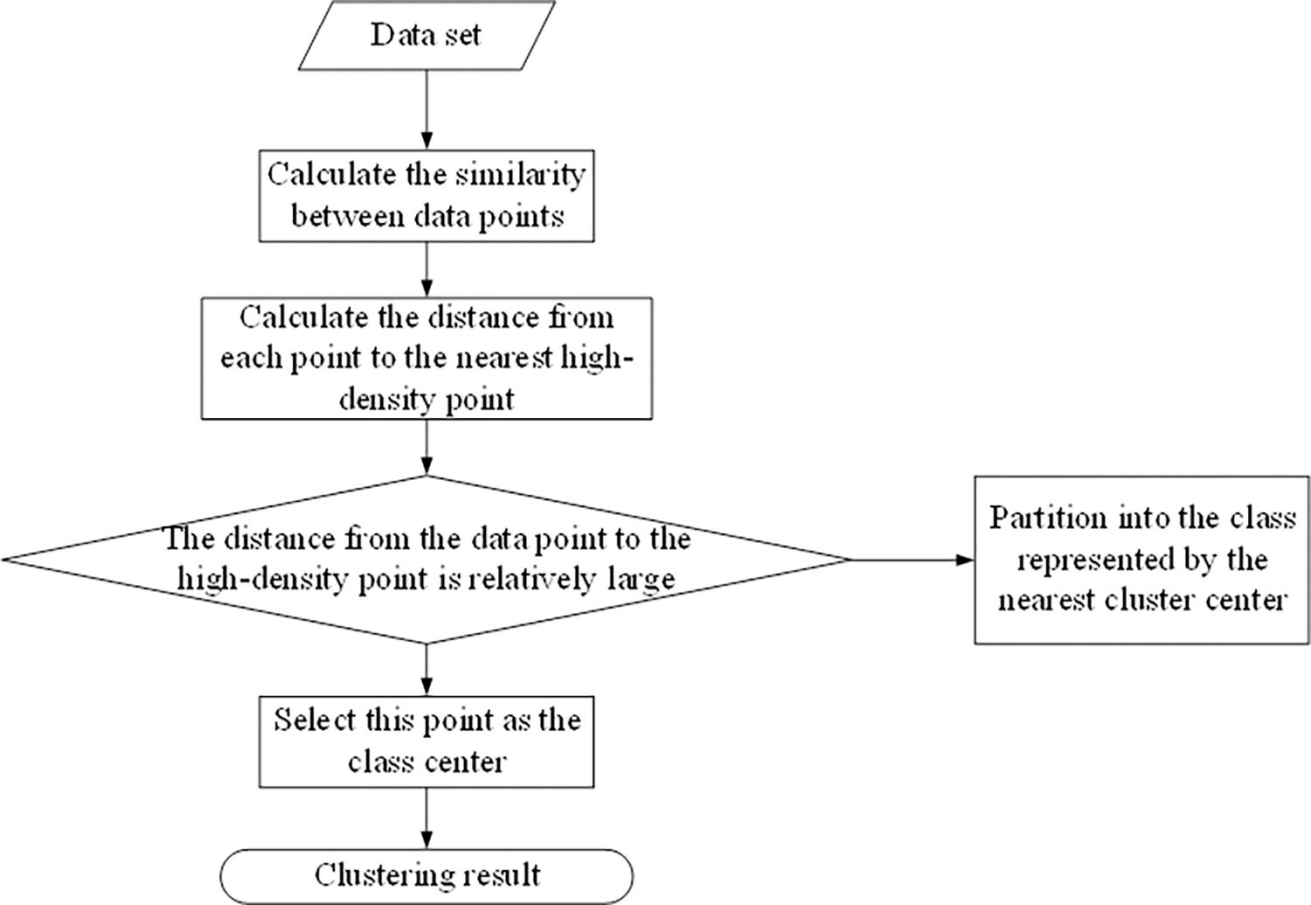
Flow chart of density peak clustering algorithm.

The final constructed higher-order hybrid clustering algorithm comprises three key components: initial data clustering, centroid acquisition, and class merging. The PSO clustering algorithm initially engages in data clustering, amalgamating multiple class clusters from datasets after a finite number of iterations. Candidate centroids are derived from the data attributes via the density peak search algorithm, as illustrated in Fig 3. Subsequently, the initial class clusters and candidate centroids facilitate centroid determination. Finally, a division-based approach allocates data points to the class corresponding to the nearest centroid. Given scenarios with multiple identified class centers, a class merging operation is employed at the algorithm’s conclusion to consolidate class clusters.

After inputting the dataset to be clustered, the algorithm proceeds as follows:

Step 1: Calculate the distance between two data points *d*_*ij*_(*i*≠*j*).

Step 2: Calculate the truncation distance *d*_*c*_, where *d*_*c*_ = *d*_*f(Mt)*_, *f*(*Mt*) is obtained by rounding Mt.

Step 3: Calculate *p*_*i*_ and the distance *φ*_*i*_. We use the truncated kernel calculation, which is the common way to calculate *p*_*i*_. Thus $p_{i}=\sum_{j}\chi (d_{ij}-d_{c})$, where *χ* is defined as follows:

$$\chi (x)=\{\begin{array}{c} 1,x<0\\ 0,x\geq 0 \end{array}$$ (11)

Distance *φ*_*i*_ is measured by calculating the minimum distance between point i and any other point with a higher density:

$$\varphi_{i}=\min_{j:p_{j}>p_{i}}(d_{i,j})$$ (12)

For densely populated points:

$$\varphi_{i}=\max_{j}(d_{i,j})$$ (13)

It can be noticed that only those points whose densities are locally or globally maximal are larger than the normal neighbour spacing, i.e., there is a large *φ*_*i*_, so that points with abnormally large values of *φ*_*i*_ may also be the clustering centres. The clustering process of the algorithm is to take the points with large local density *p*_*i*_ and large *φ*_*i*_ as the class centre, and the points with small local density but large *φ*_*i*_ are considered as anomalous points. Once the class centres are identified, the other points are divided into the classes represented by the closest class centres.

Step 4: Calculate *y*_*i*_ and sort it in descending order according to the following formulae to get the set of candidate centroids *CEN*_*sus*_ = (*cen*_1_,*cen*_2_,⋯,*cen*_*s*_), *y*_*i*_ is calculated as follows:

$$y_{i}=p_{i}*\varphi_{i}$$ (14)

Step 5: Run the improved PSO algorithm from Section 3.1 to get the initial clustering of the data *Int* = (*c*_1_,*c*_2_,⋯,*c*_*n*_)

Step 6: Judge the candidate centroids of the class affiliation in the middle to get the exact set of clustered centroids *CEN*_*sure*_.

Step 7: Divide the remaining data points to their nearest class centroid to get the final class cluster.

We are in step 5, the PSO algorithm stops iterating after a certain number of iterative runs, at this point the data has formed the beginnings of the class, but not all the data is well classified into a fixed class, at this point it is also necessary to appropriate processing of the existing results. If there is no data in the neighbourhood of an object or its neighbourhood is less than a certain constant, it is considered to be an isolated point and the point is not counted in the initial clustering. Otherwise the point and its neighbourhood are considered as a class, from which multiple classes are formed to get the initial clustering *Int*.

The formation of the candidate centroid set is carried out next. The candidate centroid set is defined in the algorithm in order to eliminate the manual selection process. Firstly, calculate *y*_*i*_ = *p*_*i*_ *φ_*i*_ to get *y*_*i*_, then calculate the second-order difference value of *y*_*i*_, find the index position where the very small value point is located, and intercept the first few data points of the index position, but in most cases, the number of points intercepted by this method is larger than the real class centre, so we form the candidate centroid set here for the next steps.

In determining the centroids from the set of candidate points, we mainly applied the initial clusters formed in step 5 *Int*, which determine the centroids as follows:

For each class, we first calculate the sum of the distances S from the data points in the class to the candidate centre *cent*_*i*_, S is calculated as follows:

$$S=\sum_{x_{i}\in c_{i}}d(x_{i},cent_{i})$$ (15)

The candidate centroid with the smallest S is subsequently used as the exact centroid.

## Experiments and analysis

To evaluate the effectiveness of the proposed methodologies, we conducted experiments using one synthetic dataset, S1, and the 1-gram UCI real dataset CMC. The dataset parameters are detailed in Table 1. Our comparative analysis includes K-means [4], HPSOK-Means [13], and PSC-RCE [18]. HPSOK-Means utilizes the PSO algorithm to determine the centroids of K clusters, initializing particles using the clustering outcomes from K-means. PSC-RCE is a notable particle swarm clustering algorithm, streamlining particle swarm clustering update rules to significantly reduce computational overhead by enhancing trajectory efficiency.

**Table 1:** Data set parameters.

	Data set	Scale	Number of features	Number of clusters
Synthetic data set	S_1_	1200	2	4
Real data set	CMC	1473	9	3

To ensure fair comparisons, the maximum fitness evaluations for the PSO-based clustering algorithm are capped at 3000, while K-Means’ maximum objective function calculations are set to 30000 without additional parameters. Other parameters for the comparison algorithms, excluding K-means, align with their respective original papers. To mitigate experimental bias, each algorithm underwent 30 independent runs on each dataset.

### Experimental indicators

The F-measure serves as a pivotal metric for evaluating clustering performance comprehensively, amalgamating precision and recall as vital indicators. Within clustering analysis, the F-measure value directly mirrors the efficacy of the clustering outcome.

Precision primarily quantifies the fraction of positive example samples correctly identified within the clustering results, indicating the ratio of true examples to the samples predicted as positive examples.

Conversely, Recall assesses the proportion of all actual positive example samples correctly identified, representing the ratio of true examples to the total number of actual positive example samples.

Formally, each benchmark classification *C*_*i*_ (given by the true labels of the input dataset) corresponds to a collection of *n*_*i*_ objects required for a query. Each cluster *C_j_*' obtained by the clustering algorithm corresponds to the set of *n*_*j*_ objects retrieved from a query. *n*_*ij*_ denotes the number of objects in the base classification *C*_*i*_ in the cluster *C_j_*'. For each benchmark classification *C_j_*' and cluster *C_j_*', F-measure(F), Precision and Recall are defined as follows:

$$\mathrm{Precision}(i,j)=\frac{n_{ij}}{n_{j}}$$ (16)

Recall(i,j)=nijni (17)

$$F(i,j)=\frac{\left(b^{2}+1)\times P(i,j)\times R(i,j\right)}{b^{2}\times P(i,j)+R(i,j)}$$ (18)

$$F=\sum_{i}\frac{n_{i}}{N}\times \max_{j}\{F(i,j)\}$$ (19)

where b in Eq (18) is equal to 1, which allows both precision and recall to remain equally weighted.

### Results

**Fig 4:**
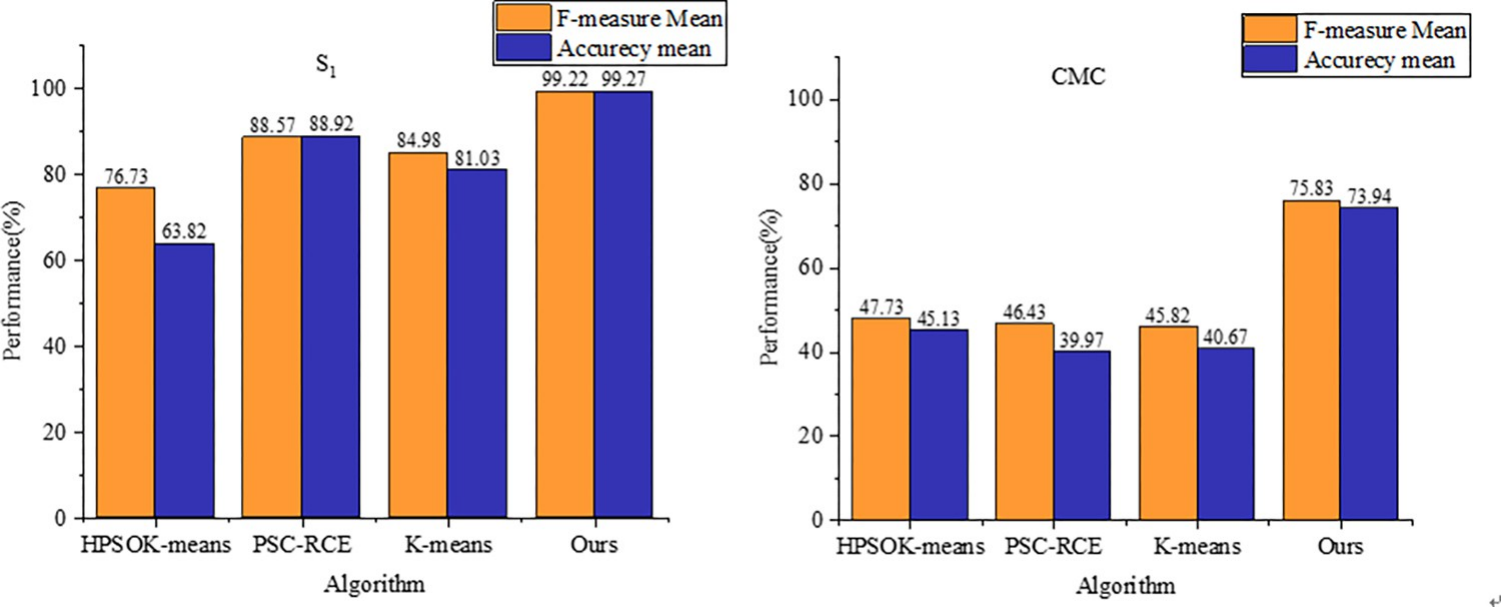
Experimental comparison of different clustering algorithms.

In order to comprehensively assess the performance and stability of the algorithms presented in this paper alongside the comparative algorithms, this subsection presents experimental comparison results across both datasets. Fig 4 illustrates the average F-measure and recall values for each scheme on datasets S1 and CMC. The algorithm introduced in this paper exhibits superior performance compared to the three comparative algorithms. Notably, on dataset S1, this paper’s algorithm attains an average F-measure and recall above 99%, surpassing all others. The best-performing PSC-RCE algorithm achieves only 88.57% and 88.92% for F-measure and recall, respectively, substantially trailing behind this paper’s scheme by 10.65% and 10.35%.

Furthermore, the experimental results obtained by the proposed model display a highly concentrated overall distribution with minimal outliers. This observation indicates robust stability across both datasets, affirming the strength of this paper’s algorithm.

**Fig 5:**
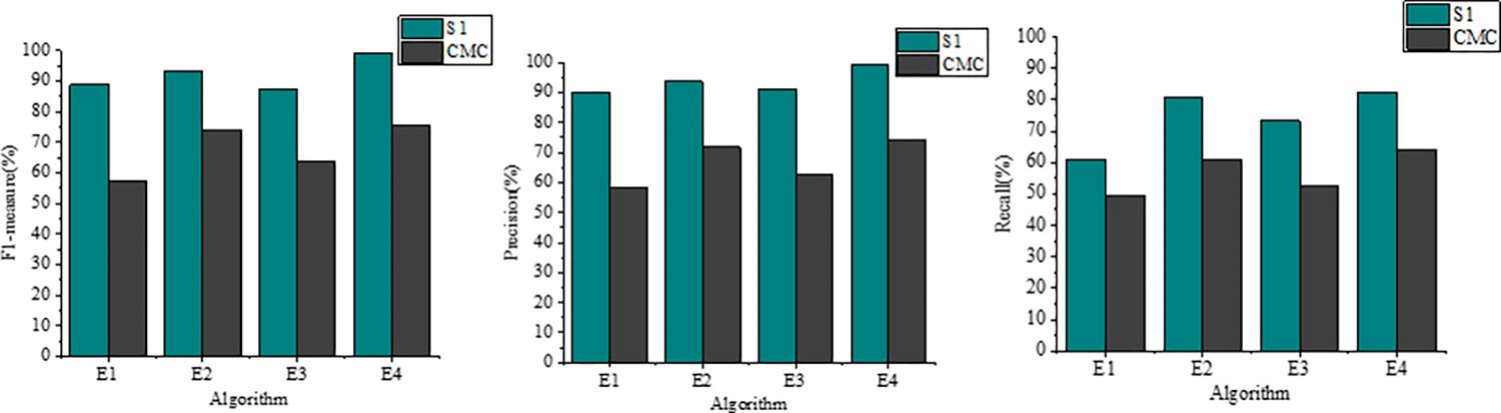
Results of ablation experiment.

To scrutinize the stability factors behind the algorithms presented in this paper, we conducted ablation experiments, considering variations such as the original PSO algorithm, the improved PSO algorithm, and the inclusion or exclusion of density values for clustering. We denoted E1 as the clustering algorithm devoid of density values and lacking the improved PSO algorithm, E2 as the clustering algorithm devoid of density values but integrating the improved PSO algorithm, E3 as the clustering algorithm incorporating density values without the improved PSO algorithm, and E4 as the clustering algorithm integrating both density values and the improved PSO algorithm. Experimental evaluations of F-measure, recall, and precision for each model are depicted in Fig 5.

Comparing E2 with E1 highlights a substantial enhancement in F-measure, precision, and recall (73.91%, 71.91%, and 60.91%, respectively, on dataset CMC) upon employing the improved PSO algorithm. Relative to E3 and E1, it’s evident that introducing density values without a higher-order clustering algorithm doesn’t efficiently enhance performance. Moreover, on dataset S1, the recall values for E1, E2, E3, and E4 reach 60.92%, 80.62%, 73.23%, and 82.22%, respectively. These metrics underscore that the higher-order hybrid clustering algorithm devised in this paper augments traditional PSO clustering algorithm performance by approximately 34.96%.

**Fig 6:**
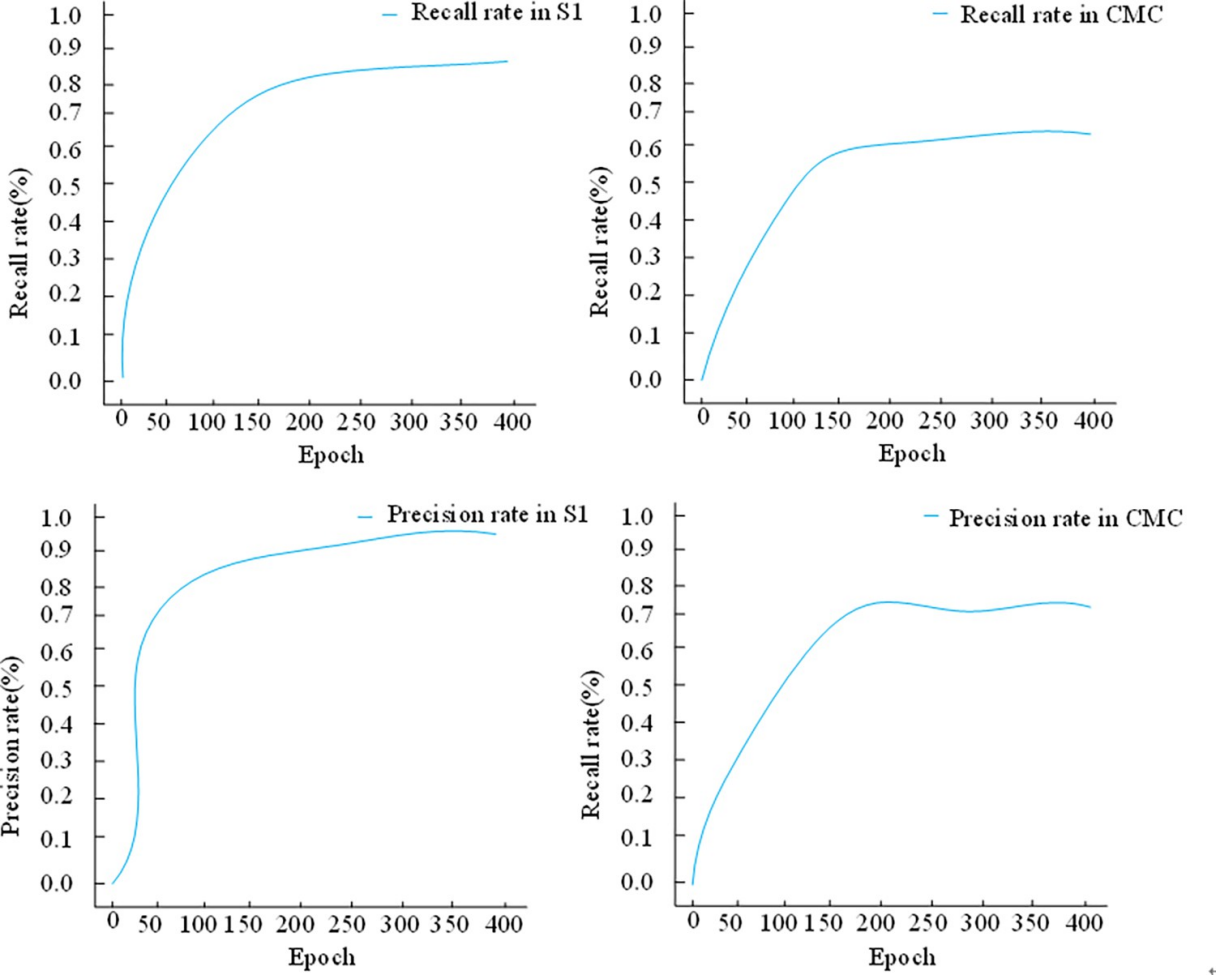
Training process.

Fig 6 illustrates the iterative training’s impact on the clustering algorithm’s ability to identify truly positive samples, as measured by recall and precision metrics. Initially, as the algorithm learns, it gradually identifies more truly positive samples, leading to a steady increase in both recall and precision. Notably, due to our mitigation of overfitting concerns, recall and precision remain steady without declining over the course of iterations.

Moreover, the relatively balanced distribution of positive and negative samples in both datasets, coupled with the algorithm’s high stability, results in moderate fluctuations in recall and precision. Ultimately, the recall stabilizes at 82.22% and 62.87%, while precision stabilizes at 98.89% and 73.87% across both datasets.

**Fig 7:**
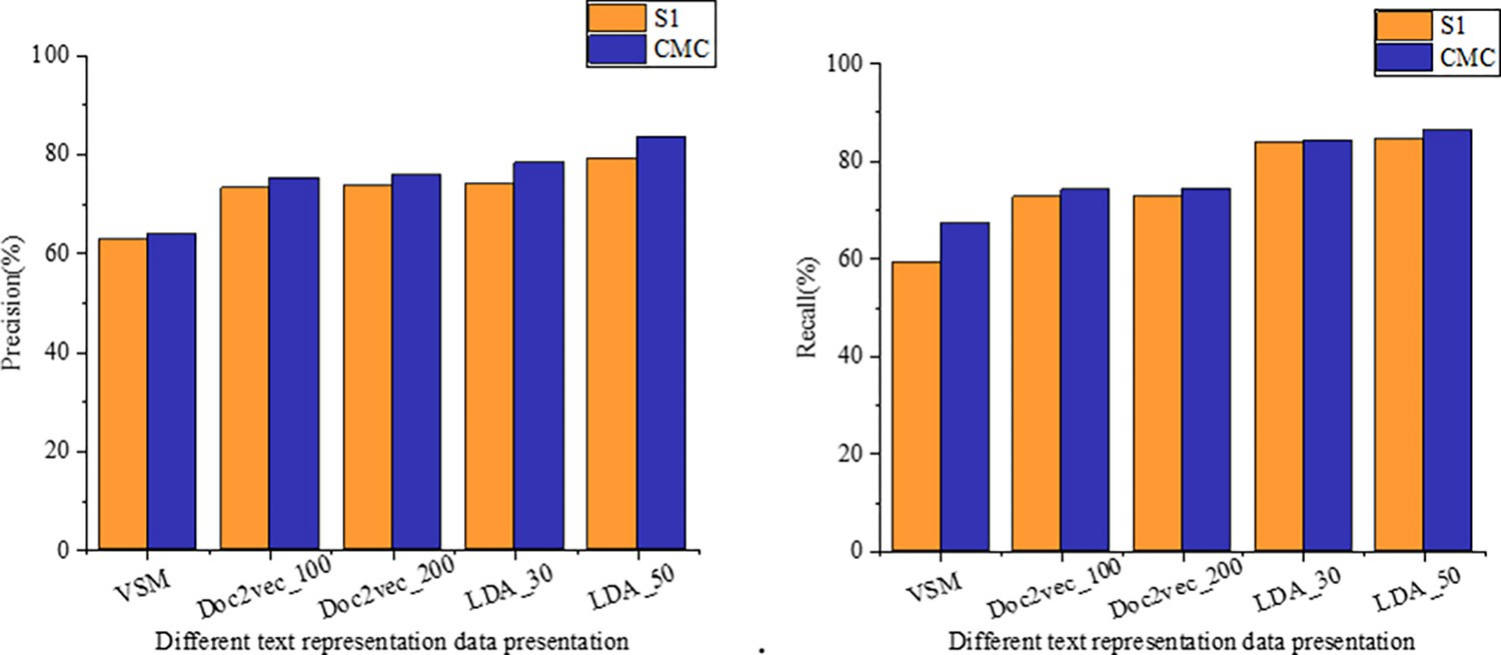
Comparison of clustering performance under different text vector formation methods.

To further explore the efficacy of the algorithm presented in this study for text data clustering, we conducted experiments on synthetic datasets employing various text vectorization methods. This investigation aimed to ascertain the algorithm’s consistency across diverse text vector representations and identify the representations that optimize its performance. Fig 7 illustrates the impact of the algorithm on both synthetic and CMC datasets, depicting accuracy and recall concerning Doc2vec and LDA subject models. Given the flexibility to train vectors to varying dimensions, our experimentation with Doc2vec involved dimensions of 100 and 200, while LDA utilized dimensions of 30 and 50. The VSM dimension corresponds to the lexicon, encompassing 75,307 feature items.

Observing minimal differences in clustering outcomes across varying dimensions of the same representation, it’s evident that LDA, leveraging thematic vectors, crafts semantic-level textual representations, yielding the highest accuracy and recall post-clustering. Specifically, employing LDA thematic vectors with a dimensionality of 50, this paper’s approach achieves an accuracy rate of 79.2% and 83.5% on datasets S1 and CMC, respectively, alongside recall rates of 84.4% and 86.2%. These rates notably surpass other text data representations.

Contrarily, while LDA demonstrates superior performance, few studies adopt LDA topic vectors for text vectorization. To address this, we introduced Doc2vec, a document representation model necessitating iterative runs. Results indicate that with Doc2vec dimensions set at 100, this paper’s approach achieves accuracies of 73.1% and 75.1% on datasets S1 and CMC, respectively. With Doc2vec dimensions set at 200, accuracies reach 73.6% and 75.8% on the same datasets. Notably akin to the LDA model, Doc2vec generates text vectors at the semantic level via neural network-based formation, yet it lacks interpretability, warranting further investigation.

Furthermore, our findings underscore VSM’s notably inferior results. This can be attributed to VSM employing lexical items as vector dimensions and TFIDF features as word weights, resulting in excessively high-dimensional and sparse representations, thereby exhibiting poor algorithmic performance.

**Fig 8:**
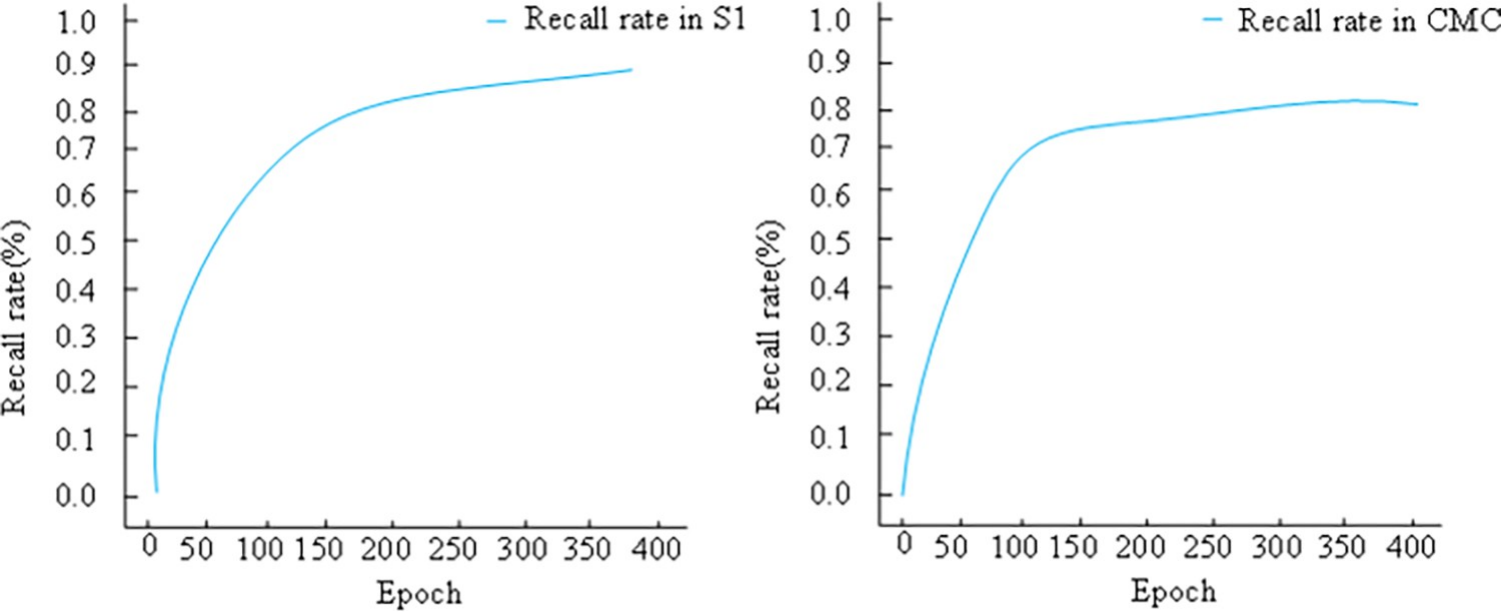
Recall curve of higher-order hybrid clustering algorithm incorporating LDA model.

The LDA topic model’s dimensionality reduction yields a more concise and meaningful data representation, capturing semantic information within documents. This enhancement aids clustering algorithms in comprehending document content more effectively. Iterative experiments conducted on the higher-order hybrid clustering algorithm (Fig 8) reveal that employing LDA’s dimensionality reduction enables accurate clustering of similar data, enhancing clustering quality to achieve stability with fewer iterations.

Remarkably, on dataset S1, after 250 iterations, a rate of 93.41% is achieved, while on dataset CMC, 79.85% is reached at 130 iterations. This notably enhances cluster recall. Consequently, integrating the LDA Topic Vector Model into the design of health information management models for the elderly promises improved data input and representation for clustering algorithms, thereby enhancing clustering efficacy and efficiency.

## Discussion

By integrating the perspective of sports and medicine, this paper successfully incorporates GEDM into the EDA of the PSO algorithm, effectively improving the convergence speed of PSO and avoiding local optima. Additionally, a high-order hybrid clustering algorithm combines the improved PSO clustering with the density peak search algorithm’s candidate centroids, optimizing solution performance. Our analysis highlights the outstanding accuracy and stability of this hybrid algorithm across various datasets.

Using the LDA topic model’s vector representation in text data enhances the performance of text data clustering by revealing latent topic information through dimensionality reduction. The high-order hybrid clustering algorithm, based on density values and the improved PSO algorithm, enables the grouping of similar data points in elderly health data, further revealing patterns in the data. Employing LDA topic vectors for text representation maximizes their advantages in dimensionality reduction, semantic richness, sparse handling, interpretability, flexibility, and scalability. This significantly improves the data representation of elderly health data clustering, enhancing clustering effectiveness and efficiency.

Integrating the perspectives of sports and medicine, constructing a high-order hybrid clustering algorithm holds important practical significance in understanding the health status of the elderly and identifying potential health issues. This comprehensive approach not only aids in a profound understanding of the physiological and movement characteristics of the elderly but also provides more comprehensive and accurate health assessments. Firstly, by integrating physiological indicators from the medical field and activity data from the sports domain, the algorithm can more comprehensively depict an individual’s health, providing healthcare professionals with more information and clues.

Secondly, the application of the high-order hybrid clustering algorithm can effectively identify potential health issues in the elderly population. By combining medical and sports data, the algorithm can identify potential health risks and signs of diseases, assisting healthcare personnel in early-stage intervention and management. This ability to detect potential health issues early has a significant positive impact on improving the quality of life and extending healthy lifespans for the elderly.

Moreover, the integrated perspective of sports and medicine can also provide a scientific basis for developing personalized rehabilitation plans and health management strategies. By understanding the movement characteristics and physiological conditions of the elderly, medical teams can design rehabilitation plans tailored to each individual’s specific needs, thereby improving rehabilitation outcomes and quality of life.

Therefore, by integrating the perspective of sports and medicine through the high-order hybrid clustering algorithm, it not only aids in a comprehensive understanding of the health status of the elderly but also provides a scientific basis for personalized healthcare and rehabilitation, thus holding important practical application prospects in the field of elderly health management.

## Conclusion

In this paper, we devised a higher-order hybrid clustering algorithm integrating peak density and PSO methodologies to address geriatric health information management within sports integration. Enhancements to the PSO algorithm involve integrating GEDM into EDA, accelerating convergence and preventing local optima. In the initial clustering phase of our hybrid algorithm, based on density values and the improved PSO, we merge class clusters and candidate centroids to determine final centroids. Our experiments on datasets S1 and CMC demonstrate remarkable algorithm performance. Specifically, this paper’s algorithm achieves outstanding F-measure, recall, and precision of 99.13%, 82.22%, and 99.22% on dataset S1 and 75.22%, 64.22%, and 74.22% on dataset CMC, surpassing comparison schemes significantly. Moreover, leveraging the text vector representation of the LDA topic vector model facilitates efficient dimensionality reduction, particularly notable when the LDA topic vector dimension is set to 50. Ultimately, our higher-order hybrid clustering algorithm attains accuracy rates of 79.2% and 83.5%, alongside recall rates of 84.4% and 86.2% on datasets S1 and CMC, respectively. Consequently, within elderly health information management, employing text vector representation with LDA topic vectors significantly bolsters health data clustering performance. It expedites the detection of elderly health data, enabling swift and efficient formulation of corresponding treatment plans, thereby advancing the convergence of body and medicine integration.
